# Coloration in Equine: Overview of Candidate Genes Associated with Coat Color Phenotypes

**DOI:** 10.3390/ani14121802

**Published:** 2024-06-17

**Authors:** Xiaotong Liu, Yongdong Peng, Xinhao Zhang, Xinrui Wang, Wenting Chen, Xiyan Kou, Huili Liang, Wei Ren, Muhammad Zahoor Khan, Changfa Wang

**Affiliations:** Liaocheng Research Institute of Donkey High-Efficiency Breeding and Ecological Feeding, Liaocheng University, Liaocheng 522000, China

**Keywords:** equine, coat color, melanin, pigmentation, candidate genes

## Abstract

**Simple Summary:**

Color and body size traits are considered the key parameters influence the economic value of animals. In recent years, advancement in the genetic basis of coat colors in equines has received considerable attention among animal breeders. In addition, coat color plays a significant role in breed identification and selection, as well as animal health and disease. The current review concisely provides information on the role of melanin pigments and key candidate genes associated with coat color phenotypes in equines. Furthermore, the review also highlights the importance of coat color in equine breeding and health.

**Abstract:**

Variation in coat color among equids has attracted significant interest in genetics and breeding research. The range of colors is primarily determined by the type, concentration, and distribution of melanin pigments, with the balance between eumelanin and pheomelanin influenced by numerous genetic factors. Advances in genomic and sequencing technologies have enabled the identification of several candidate genes that influence coat color, thereby clarifying the genetic basis of these diverse phenotypes. In this review, we concisely categorize coat coloration in horses and donkeys, focusing on the biosynthesis and types of melanin involved in pigmentation. Moreover, we highlight the regulatory roles of some key candidate genes, such as *MC1R*, *TYR*, *MITF*, *ASIP*, and *KIT*, in coat color variation. Moreover, the review explores how coat color relates to selective breeding and specific equine diseases, offering valuable insights for developing breeding strategies that enhance both the esthetic and health aspects of equine species.

## 1. Introduction

Historically, horses and donkeys played a crucial role in facilitating inter-regional trade, as they were utilized for transportation and labor [1,2,3]. China’s horse industry has experienced significant growth due to the nation’s rapid socio-economic advancements and improved living standards. As a result, equestrian sports have gained widespread popularity as a recreational pursuit throughout the country. At the same time, the donkey industry has emerged as a significant source of income for farmers and herdsmen, driven by the increasing demand for donkey-derived products such as meat, milk, colla corii asini, and various specialty items.

Horses and donkeys were domesticated around 6000 years ago [4,5,6]. The domestication process is believed to have been complex and multi-staged, resulting in changes in the movement, morphology, and physiology of these animals compared to their wild ancestors [7,8,9]. One of the prominent features reflecting these changes is coat color. Prior to domestication, wild populations exhibited fewer variations in coat color. Different coat colors may have played roles in activities such as camouflage, mating behavior, and predation [10,11], which influenced survival and reproductive success. However, through natural adaptation and human-mediated selective breeding, there has been a gradual diversification of coat color phenotypes. Therefore, to fully understand a specific breed, it is necessary to understand the genetic basis underlying its coat coloration.

Coat color is a visually recognizable phenotypic trait that follows Mendelian laws of inheritance [12,13]. It has attracted widespread attention from scholars worldwide and has become a research focus in the field of genomics. As living standards rise, rare colors become more desirable, especially for ornamental or companion animals [14,15]. Coat color also plays a significant role in the purchasing decisions of buyers, with biases against certain colors influencing the selling price of the animal [16,17]. Identifying horse breeds and breed registration often rely on coat color as a crucial factor. Certain criteria and limitations must be met for horses to be registered, such as the American Paint horse, Appaloosa horse, and Pura Raza Español (PRE) horse [18].

Coat color is also a significant factor in breed selection. Researchers have used gene editing techniques to develop Duroc pigs with black coats, aiming to create superior breeds with high growth rates, high meat production, and coat color preferences of consumers [19]. Understanding the genetic mechanisms behind coat color diversity is crucial for assessing breeding germplasm resources. Previous studies have investigated the molecular basis of coat color traits in breeding animals. They used methods such as genetic variation analysis and differential gene expression analysis to study coat color genes associated with different coat color phenotypes within the same species [20,21]. Coat color can provide insights into an animal’s health and disease status, as genetic variations controlling coat color can be associated with various diseases. Previous research has connected coat color with conditions such as deafness, eye disease, albinism, melanoma, and other ailments in animals [22,23,24,25,26,27]. In recent years, progress has been made in studying the genetic markers associated with coat color in various livestock animals. Table 1 summarizes the genes associated with coat color phenotypes in animals.

The intersection of two important trends, the significance of coat color markers in livestock selection and the advancement of genomics technologies, necessitates a comprehensive review of current research on coat color traits. Therefore, this review paper aims to clarify the value and biological implications of genetic regulation mechanisms for coat color, with a specific focus on its relevance to disease and potential in selective breeding. In particular, it examines the expression mechanisms of major candidate genes for coat color in horses and donkeys. Additionally, it evaluates the regulatory mechanisms of genes involved in coat color control within biological pathways and investigates the relationship between coat color and the health of horses and donkeys. By doing so, it aims to contribute to our understanding of equids and establish a scientific basis for future endeavors in selective breeding.

## 2. Coat Color Classification in Equines

### 2.1. Coat Color Classification in Horses

Horses exhibit a variety of coat colors that can be classified using different criteria. Consistently, Lauvergne et al. [47] proposed four dimensions for describing horse coat color. These dimensions include pigmentary pattern, eumelanic type, pigmentation alteration, and white designs, covering the entire range of variability. Furthermore, in *Equine Color Genetics*, 4th Edition, Sponenberg et al. [48] describes the fundamental colors of horses (bay, chestnut, black, and brown) and then explains dilutions and white patterning based on the genetic foundations of these underlying coat colors. Similarly, Thiruvenkadan et al. [49] classified horse coat colors into three categories: basic colors (black, bay, and chestnut), diluted colors (cream, dun, silver dapple, and champagne), and white base colors (determined by the presence of white coat mixtures such as gray, pinto, and white) or white patches.

(1) Black: The entire coat is primarily black.
(2) Bay: The coat is brown with black points and shades.
(3) Chestnut: The coat color is varying shades of red and have non-black points.
(4) Diluted colors: The coat color becomes lighter and sometimes nearly white.
(5) White spotting: The patterns of white spotting are added independently to any colored coat.

### 2.2. Coat Color Classification in Donkey

In comparison to horses, donkeys have less variation in coat color and can be divided into the following main categories [48]:

(1) Black: The entire coat is primarily black.
(2) Sanfen: The entire coat is black, with white fur around the mouth, eyes, and underbelly.
(3) Gray: The coat is predominantly greenish-gray, greenish-brown, or greenish-white, with lighter coloring on the belly and nose. The long hairs are black or nearly black.
(4) Cyan: Mixed black and white hairs cover the body, and the number of white hairs increases with age.
(5) Chestnut: The coat is mostly red all over the body, with lighter, almost white coloring around the mouth, nose, eyes, underbelly, and inside the limbs.
(6) White: The entire coat is white or light gray.

## 3. Mechanisms of Pigmentation

The formation of hair color is determined by the structure of the hair follicle, which is a complex skin appendage resulting from the interaction of the epidermis and dermis [50,51]. The hair follicle consists of various cells, including melanocytes, Merkel cells, and Langerhans cells, each serving different functions such as pigmentation, induction, and immune response [52,53]. What sets the hair follicle apart is its cyclic growth, which involves rapid growth (anagen), apoptosis-driven regression (catagen), and a quiescence phase (telogen) [50,51,52,53].

The variety of coat colors in animals is influenced by multiple factors. Different species show different coat colors, and even within the same species, there is a significant range of coat colors. Mammalian coat color variation is affected by the type, distribution, and content of melanin [54,55]. Melanin is produced by melanocytes (MCs), which derive from melanocyte stem cells (McSCs) originating from the neural crest. These cells are located in the bulge of the hair follicle alongside hair follicle stem cells (HFSCs). During the anagen phase, both HFSCs and McSCs are activated. HFSCs generate new hair follicles, while McSCs migrate to the bulb and differentiate to produce MCs, which, in turn, produce melanin and color the hair [56,57]. In the catagen phase, MCs undergo apoptosis, leading to a cessation of melanin synthesis in the hair [52,58]. During the telogen phase, both HFSCs and McSCs remain relatively inactive, awaiting the next hair cycle [58,59].

**Figure 1:**
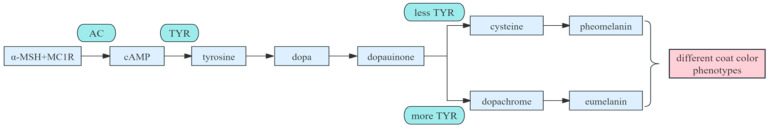
Pathway of melanin synthesis.

Melanin can be classified into two types: eumelanin, which comprises brown to black particles, and pheomelanin, which comprises yellowish to slightly reddish particles [60,61,62]. The ratio of these two types determines the hair color differentiation. The melanin synthesis pathway is depicted in Figure 1. Notably, α-MSH binds to MC1R, activating adenylate cyclase (AC). This activation leads to an increase in intracellular cAMP levels and subsequent tyrosinase activation [63]. Tyrosinase, in turn, catalyzes the conversion of tyrosine to dopa, which is further oxidized to dopaquinone. Dopaquinone serves as a precursor for both true melanin and fucoxanthin synthesis. In the presence of cysteine, dopaquinone reacts to form fucoxanthin. Conversely, in the absence of cysteine, dopaquinone undergoes intramolecular cyclization, generating cyclodopa, which then leads to the formation of dopa pigment. The decomposition products of dopa pigment are oxidized to produce eumelanin [21,64,65,66]. Additionally, the regulation of melanin levels is influenced by the tyrosine family, which includes tyrosinase (*TYR*), tyrosinase-related protein-1 (*TYRP 1*), and tyrosinase-related protein-2 (*TYRP 2*), also known as dopachrome tautomerase (*DCT*) [67,68].

## 4. Candidate Genes Associated with Coat Color Phenotypes

**Figure 2:**
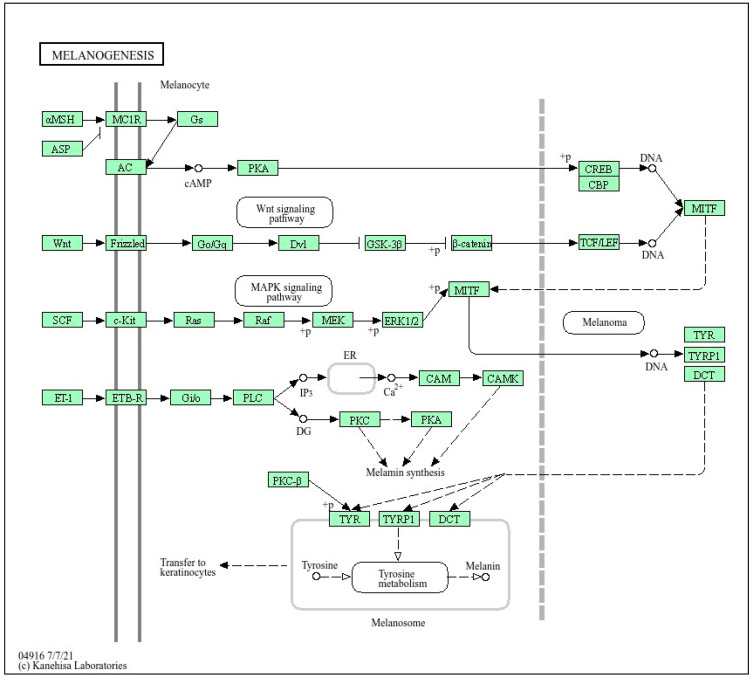
Mechanism of regulation of the melanogenesis signaling pathway (eai04916) by coat-color-linked genes (*MC1R*, *TYR*, *MITF*, *ASIP*, and *KIT*).

In recent years, extensive research has been conducted by scholars both domestically and internationally on the genetics of equine coat color, resulting in significant progress. The formation of the majority of coat colors can be reasonably explained, with reported genes including *MC1R*, *ASIP*, *TYR*, *MITF*, *KIT*, *EDNRB*, *STX17*, *MATP*, and *PMEL17.* Furthermore, these genes have been extensively documented for their critical role in coat colors in horses and donkeys. Using DAVID analysis, it was revealed that these genes are significantly involved in regulation of the melanogenesis signaling pathway, which has a critical role in the synthesis of melanin pigments (eai04916), as shown in Figure 2.

### 4.1. Candidate Genes Associated with Coat Color Phenotypes in Horses

#### 4.1.1. MC1R and ASIP

The primary horse coat colors are black, chestnut, and bay, with their expression largely influenced by two key coat color candidate genes, *MC1R* and *ASIP* [69,70]. The correlation between their alleles and resulting phenotypes is illustrated in Table 2. Additionally, Reissmann et al. [71] observed that these three fundamental coat colors were prevalent across nearly all horse breeds, as evidenced by their analysis of coat color alleles in 1093 horses from 55 different breeds and 20 Przewalski’s wild horses. Furthermore, Mura et al. [72] discovered that the predominant coat colors were black, chestnut, and bay, with no presence of spotting or color dilution by analyzing the genetic distribution of coat color in 90 Sarcidano horses.

*MC1R* is a G-protein-coupled receptor expressed in the skin and melanocytes [73]. The *MC1R* gene, located on equine chromosome 3, consists of exons that are 954 bp long [69]. When MC1R has the dominant allele (EE = E), it promotes the production of true melanin, resulting in a black coat color. Conversely, a mutated *MC1R* gene that produces a recessive allele (Ee = e) promotes the production of fucoxanthin, leading to a chestnut coat color [69,70]. For example, Marklund et al. [28] identified a missense mutation (TCC-TTC) in the *MC1R* gene, causing codon 83 to change from serine to phenylalanine, ultimately resulting in the chestnut phenotype. The *ASIP* gene is located on horse chromosome 22. In the case of *ASIP,* the dominant allele (AA = A) encodes a protein that competes with α-melanocyte-stimulating hormone (α-MSH) for binding to *MC1R.* This competition inhibits the production of eumelanin, and instead promotes the synthesis of pheomelanin [69,74,75]. Accordingly, Rieder et al. [30] discovered an 11 bp deletion in exon 2 of *ASIP*, leading to a recessive allele (Aa = a) that is unable to code for the proteins necessary for eumelanin synthesis, thus being associated with recessive black coat color in horses. However, subsequent to the availability of the EquCab3.0 sequence, it was proposed that the mutation actually occurred in exon 3 [69]. Furthermore, Wagner et al. [76] identified a novel allele, ea, with a mutation at codon 84 (GAC-AAC), resulting in a change from aspartic acid to asparagine. The phenotype of chestnut hairs resulting from this mutation closely resembles the general chestnut hair phenotype, with variations in shade based on different combinations of (*e*/*e*), (*e*/*ea*), and (*ea*/*ea*) genotypes. There is also speculation that intergenic regulatory variation may enhance *ASIP* gene expression, leading to a redder coat color and a brighter bay appearance in horses [77].

#### 4.1.2. KIT

The proto-oncogene c-kit is located on chromosome 3 in horses and affects the survival, migration, and differentiation of melanocytes. It is a major candidate gene for the formation of white coat color [78,79,80]. Previous studies have shown that four white coat color traits, Dominant White, Tobiano, Sabino, and Roan, are associated with the *KIT* gene, as depicted in Table 3.

Patterson et al. [81] identified a new mutation, W34, that is significantly associated with white markings in 147 horses. This mutation involves replacing a polar threonine encoded by the *KIT* gene with a nonpolar alanine. Additionally, this variant interacts with W19. Interestingly, McFadden et al. [82] clarified that the W35 allele is caused by a single nucleotide mutation in the 5′ untranslated region. Having at least one copy of this allele can affect the migration of melanocytes, resulting in an increase in white spots on the coat. Furthermore, Hug et al. [83] detected a heterozygous 1273 bp deletion that spans parts of intron 2 and exon 3 of the *KIT* gene in the whole-genome sequence data of the German Riding Pony, defining this mutation as W28. Similarly, Patterson et al. [84] identified and confirmed two variants in the white spotted horse family. The first variant, W31, is a leucine to tyrosine shifter mutation in exon 1 of the *KIT* gene, leading to an early termination codon and shortening of the normal amino acid sequence. The second variant is a mild white spotting pattern caused by the W32 non-synonymous SNP. Consequently, Brooks et al. [85] discovered that the C to G base substitution in intron 13 of the *KIT* gene does not directly cause the Tobiano coat color. However, the association between Tobiano and this polymorphism in the *KIT* gene provides further evidence for *KIT* as a potential gene related to Tobiano coat color. Subsequent research revealed that the Tobiano coat color is actually the result of a paracentric inversion of horse chromosome 3, although this inversion does not directly affect the structure of the *KIT* gene. This finding may help explain the appearance of the cryptic Tobiano phenotype [36,86]. Similarly, Brooks et al. [80] identified a single nucleotide polymorphism resulting from a T and A base substitution in intron 16 and a subsequent deletion in exon 17, leading to the formation of Sabino 1 (SB1). However, this specific polymorphism cannot fully account for the various Sabino types observed. In a study by Voß et al. [87], the exons of the *KIT* gene were sequenced in Roan and non-Roan Icelandic horses, revealing eight variants significantly correlated with heterozygous coloration. Of note, the insertion in intron 13 (ECA 3: 79, 548, 356) showed the strongest association (*p*-value = 0.0002), suggesting a potential relationship between the *KIT* gene and the Roan coat color phenotype in Icelandic horses.

#### 4.1.3. Diluted Genes and Their Association with Coat Colors

The *SLC45A2* gene, located on chromosome 21 in horses [88], encodes a transmembrane protein in melanocytes that plays a role in transporting molecules necessary for the proper functioning of melanosomes [42]. Mutations in this gene can lead to a dilution phenotype in coat color. Correspondingly, Holl et al. [43] identified the missense variant *SLC45A2:* c.985G>A as responsible for the pearly coat color allele *Cprl,* while a new allele (Csun) was linked to the missense variant *SLC45A2:* c.568G>A, resulting in coat color pigmentation dilution. In a recent study, Sevane et al. [89] found that a polymorphism c.985G>A in exon 4 of *SLC45A2* was significantly associated with the pearl/cream phenotype, suggesting that selecting for the *Cprl* gene could produce pearl hair color phenotypes. Correspondingly, Bisbee et al. [42] discovered that the variant c.305G>A in *SLC45A2* was only present in purebred mares among 15 Gypsy horses, leading to a dilution phenotype named snowdrop (Csno). This dilution phenotype is similar to cream dilution, and possibly has no effect on heterozygotes.

The Dun phenotype is identified by its lightened coat color and dark markings [45]. Imsland et al. [45] revealed that the Dun phenotype is linked to the normal expression of *TBX3* on equine chromosome 8, and that a 1617 bp deletion mutation in the *TBX3* gene leads to a non-dun coat color. Additionally, another study indicated that the *TBX3* 1.6 kb insertion–deletion in the haploid plays a significant role in determining the degree of dilution in the phenotype [90].

Silver dapple is a result of melanin dilution, particularly in the long hairs of the mane and tail, leading to white and gray coloring [46]. Correspondingly, Brunberg et al. [46] identified a significant genetic linkage between the *PMEL17* gene on equine chromosome 6 and the silver phenotype in horses. They pinpointed a missense mutation in exon 11 and intron 9 of the *PMEL17* gene as the cause of the silvery coat color. Furthermore, Reissmann et al. [34] suggested that the *SILV* gene may also play a role in the silver coat color of horses. By sequencing the *SILV* gene in 24 horses, they discovered two SNPs (g.697A>T, g.1457C>T) that were strongly associated with the silver phenotype. Notably, Cook et al. [44] reported, for the first time, that a missense mutation (c.188C>G) in exon 2 of the *SLC36A1* gene on equine chromosome 14 is the causative factor for the champagne phenotype in horses. Their findings were supported by experimental evidence showing the presence of the variant in 85 horses with the champagne-diluted phenotype and its absence in 97 horses lacking the champagne phenotype.

#### 4.1.4. Other Genes

Horses with the gray phenotypic mutation experience a gradual whitening of their hair as they age. In line with this, Rosengren et al. [41] identified a 4.6 kb duplication in intron 6 of the *STX17* gene on equine chromosome 25 as the cause of this mutation within a 352 kb region. Similarly, Carl-Johan et al. [91] revealed that the graying locus dosage affects the speed at which Lipizzan horses gray. Specifically, horses with a G1/G1 genotype do not gray, G1/G2 genotype gray slowly, G1/G3 genotype gray quickly, and G3/G3 genotype gray very quickly. In contrast to previous studies linking the gray phenotype to *STX17* intron duplication, Nowacka-Woszuk et al. [92] conducted a copy number analysis using droplet digital PCR and found that tested individuals had either six or four copies (gray) or two copies (non-gray) of the *STX17* gene fragment.

The microphthalmia-associated transcription factor (*MITF*) is located on chromosome 16 in horses, and it affects melanocyte differentiation, leading to decreased pigmentation when the gene is mutated [93]. Table 4 summarizes the alleles and their phenotypes resulting from mutations in the *MITF* gene.

Hauswirth et al. [35] discovered two polymorphisms, SW1 and SW3, in the *MITF* gene that cause white patterning in a family of quarter horses. SW1 is caused by a 10 bp insertion in the melanocyte-specific promoter, while SW3 is due to a small deletion in exon 5. Another study identified two copy number variants of the *MITF* gene linked to the blotchy white depigmentation phenotype in American paint horses. This discovery was made both before and after Henkel et al. [94] reported a heterozygous 63 kb deletion spanning exons 6–9 of the *MITF* gene. In addition, Magdesian et al. [95] identified an 8.7 kb deletion in the 3′UTR, which includes the seventh intron to the ninth exon of the *MITF* gene. Furthermore, Patterson et al. [96] used exome sequencing to detect a three-base pair deletion leading to the loss of arginine, designating the mutant allele as SW7 according to the nomenclature of the *MITF* gene mutation responsible for white spotting. Subsequently, Bellone et al. [97] used whole-genome sequencing in combination with a candidate gene approach to pinpoint a 2.3 kb structural variant known as SW8, which is responsible for Splashed white in horses. Splashed white is characterized by extensive white patches on the legs, abdomen, and face. A previous study meticulously described a missense mutation in the *MITF*-binding structural domain linked to the Splashed white phenotype in Pura Raza Española horses. The mutant allele was named SW9 [98].

### 4.2. Candidate Genes Associated with Coat Color Phenotypes in Donkey

Compared to the wide range of coat colors seen in horses, donkeys exhibit fewer coat color options, and limited research has been conducted on their coat color candidate genes. Previous studies have primarily examined three genes: *MC1R*, *ASIP*, and *KIT*. Abitbol et al. [29] conducted a study screening four red donkeys and two bay-colored donkeys for functional variants of the *MC1R* gene. They discovered that a recessive missense c.629T>C variant was consistently linked to the inheritance of the red coat color. This finding was further confirmed when genotyping 124 donkeys, and it was found that the variant was strongly associated with the inheritance of the red coat color trait. Subsequent analysis of the *ASIP* gene in 127 donkeys from six breeds revealed a single nucleotide polymorphism (c.349T>C) in black donkeys. This polymorphism led to the substitution of the 117th amino acid, cysteine, with arginine, resulting in the black coat color trait. It was found to be recessively inherited [31]. Similarly, in a recent study by Sun et al. [32], 590 individuals across 13 donkey breeds were genotyped to identify polymorphisms in the *ASIP* gene. They discovered that the c.349T>C mutation could distinguish between pure black donkeys and non-black donkeys, aiding in breeding selection for pure black donkeys. Furthermore, Haase et al. [40] identified a missense mutation in exon 4 of the *KIT* gene (c.662A>C) in white donkeys, which was not present in their control group (solid colored and white spotted). This mutation led to the substitution of tyrosine for serine. A splicing mutation (c.1978+2T>A) was also found, which is present only in white spotted donkeys. Moreover, Wang et al. [6] compared 25 Dun donkeys with dilute gray pigmentation to 23 samples with non-dilute black or chestnut coat color. They determined that a 1 bp deletion downstream of the *TBX3* gene was responsible for the difference in coat color phenotype, increasing pigmentation in non-Dun donkeys and suggesting a change in coat color during the domestication of wild donkeys into domestic donkeys. A study performed by Utzeri et al. [33] discovered an association between albinism in Asinara white donkeys and a specific missense mutation (c.604C>G; p. His202Asp) in the *TYR* gene. Subsequently, genotyping was conducted on 17 wild Asinara white albino donkeys and 8 colored breeds or populations, totaling 65 donkeys. The results confirmed that only the Asinara albino donkeys exhibited the genotype homozygous for the mutation G/G, while the other colored donkeys had genotypes C/C or C/G.

## 5. Coat Color Genetic Applications

### 5.1. Coat Color Genetic Applications in Breeding

Coat color is crucial for identifying equine breeds and individuals, as it is a key factor in registration and census records. In equine genetic breeding, researchers and breeders prioritize the assessment of desirable traits. While coat color is a visible morphological marker, which also serves as a genetic marker. However, factors such as age, environment, and diet can complicate the accurate visual identification of coat color. Mura et al. [72] conducted a genetic analysis of the coat colors of 90 Sarcidano horses, revealing discrepancies between phenotypic and genetic data, with an error rate reaching as high as 53.4%. Similarly, Nowacka-Woszuk et al. [94] genotyped a Connemara foal as non-gray, while the official record classified it as gray. Through analyzing various documents, they discovered that the foal’s true coat color was blue and had been misclassified. In a separate study, Kim et al. [99] examined the genetic makeup of 1462 Jeju horses to understand the genes influencing coat color. They observed no significant variations in the *MC1R* and *ASIP* genes across generations, but noted significant changes over time in the TO allele and *STX17* G allele associated with Tobiano coat patterns. These findings highlight the importance of analyzing coat color genes during stallion selection and emphasize the need for genetic testing before breeding. By conducting genotyping for coat color traits, breeders can make informed decisions, ensuring accurate breed registration and facilitating the selection of individuals with desired coat color characteristics. As living standards improve and the modern horse industry develops, there is growing diversification in consumer demand, particularly in the esthetics of horse coat colors. This is especially evident in competitive equestrianism and the breeding of ornamental horses, where individuals seek horses with varied coat colors and elegant temperaments. Colla corii asini, a solid gum derived from donkey skin through decoction and concentration, holds high nutritional value and is a prized traditional Chinese medicine [100]. The finest donkey skin for this purpose is sourced from the Dezhou Wutou donkey [101]. Therefore, the selection and breeding of equine animals for specific coat colors play a crucial role in enhancing economic efficiency. In comparison to donkeys, horses possess a greater number of hair color loci, many of which exhibit a significant number of compound alleles. This complexity makes consistently producing a specific hair color through selective breeding challenging in practical breeding programs, highlighting the need for further research in this area.

### 5.2. Coat Color Genetic Applications in Diseases

#### 5.2.1. Vitiligo and Melanoma

Vitiligo-like depigmentation and melanoma are common skin conditions observed in gray horses as they age, with a prevalence of up to 80% in horses aged 15 years or older [102]. Vitiligo is caused by the loss of melanocytes in the skin, resulting in white patches among areas of hyperpigmentation [103]. On the other hand, melanomas typically appear as firm black nodules and tend to develop in regions such as the tail root, anus, genitals, and periocular areas. It is worth noting that a study by Rosengren et al. [41] demonstrated a connection between the gray phenotype in horses, associated diseases, and a 4.6 kb repeat within intron 6 of the *STX17* gene. This repeat functions as a cis-acting regulatory mutation, causing an overexpression of *STX17* and the nearby *NR4A3* gene in gray horse melanoma. Moreover, gray horses with ASIP loss-of-function mutations exhibit increased melanocortin-1 receptor signaling, resulting in a higher risk of developing melanoma. In addition, a recent study has presented compelling evidence that the DPF3 gene plays a role in melanoma development. Through a genome-wide association study (GWAS) involving 1210 horses from seven breeds, this gene has been identified as crucial in gray horses who manage a high heritable tumor load associated with melanoma [104].

#### 5.2.2. Health Risks

The white phenotype of horses has a significant influence on human culture, leaving a lasting impact on global literature and art [41]. The striking and unique white coat color, along with its high economic value, sets white horses apart from other color variations. However, the white phenotype is often associated with various health issues such as embryonic lethality, deafness, and blindness [36]. Consistently, Pulos and Hutt [105] observed a ratio of 28:15 in the offspring of four heterozygous white stallions mated with white mares, which closely aligns with the expected 2:1 ratio. This suggests that the WW genotype may be lethal during early development. A previous report conducted by Esdaile et al. [106] identified a genotype frequency of 0.0063 for the W13 allele in American Miniature horses. However, this allele was not detected in Shetland ponies, and no pure W13 purities were found. The researchers speculated that the W13 gene, responsible for the white phenotype, may be lethal in its pure form. In a separate study, Magdesian et al. [107] observed that 14 deaf American Paint horses exhibited extensive white markings on the head and limbs. Interestingly, the amount of white on the neck and trunk varied among these horses. The researchers suggested that horses with extensive head and limb markings, as well as blue eyes, were more likely to be deaf. Correspondingly, Bellone et al. [99] identified a 2.3 kb structural variant in the *MITF* gene in a Splashed white Thoroughbred stallion. Deafness was confirmed in one of his offspring, an almost all-white foal, based on BAER test results. The leopard complex spotting (LP) phenotype is attributed to a mutation in *TRPM1* that significantly impairs the normal function of *TRPM1,* consequently impacting the pigmentation and hearing problems of horses [36]. Additionally, Sandmeyer et al. [108] and Rockwell et al. [109] both noted that Appaloosa horses with the LP phenotype, characterized by depigmentation with pigmented patches, are more prone to equine recurrent uveitis (ERU) compared to other breeds. ERU is a common ocular disease that presents as an inflammatory condition in the eye. Recurrent episodes of ERU can result in complications such as cataracts, intraocular adhesions, corneal ulcers, and ultimately, blindness [110]. Moreover, donkeys with the white phenotype, such as Asinara donkeys with albinism and white coat coloration, have a white coat and skin with varying degrees of reduced pigmentation in the eyes, which can range from pink to light blue. Asinara donkeys with albinism also experience diminished vision and exhibit avoidance behavior when exposed to sunlight, as their skin becomes flushed, dry, and damaged [111].

## 6. Conclusions

The rapid development of genomics and sequencing technologies has provided a solid foundation for identifying and studying genes and mutation loci that are associated with coat color in equines. Coat color plays a crucial role in registering and distinguishing equine breeds, and it can also help in identifying diseases that are linked to specific coat colors. Additionally, the demand for unique coat colors in ornamental and competitive horses, as well as the need for donkey skin for colla corii asini production, highlights the significance of these traits in equines. Therefore, genetic research on coat color in horses and donkeys is both theoretically important and economically valuable. Numerous studies have extensively investigated the genetic basis of coat color in these animals, revealing insights into the mechanisms of coat color formation and its correlation with specific diseases. Researchers worldwide have sought to explore the potential significance of coat color phenotypes as indicators in equine breeding. Consequently, coat color phenotyping is expected to be a valuable tool for refining breeding selection and predicting diseases in horses and donkeys. Moving forward, further research on coat color genes in equines is expected to provide a more precise and scientific foundation for equid breeding, genetic diversity, disease identification, and treatment.

## Figures and Tables

**Table 1:** Overview of genes associated with coat color phenotypes in animals.

Gene	Species	Coat Color	References
MC1R	Horses	Chestnut	[28]
	Donkeys	Red	[29]
ASIP	Horses	Black	[30]
	Donkeys	Black	[31,32]
TYR	Donkeys	White	[33]
SILV	Horses	Sliver dapple	[34]
MITF	Horses	Splashed white	[35]
KIT	Horses	Dominant white	[36]
	Horses	Tobiano	[37]
	Horses	Sabino	[38]
	Horses	Roan	[39]
	Donkeys	White	[40]
STX17	Horses	Gray	[41]
SLC45A2	Horses	Snowdrop	[42]
	Horses	Pearl/cream	[43]
SLC36A1	Horses	Champagne	[44]
TBX3	Donkeys	Dun	[6]
	Horses	Dun	[45]
TRPM1	Horses	Leopard complex spotting	[36]
PMEL17	Horses	Sliver dapple	[46]

**Table 2:** Genotypes of the three basic coat colors in horses.

Coat Color	Extension Genotype	Agouti Genotype	Genotype
Black	E-	aa	EEaa/Eeaa
Bay	E-	A-	EEAA/EeAA/EEAa/EeAa
Chestnut	ee	A-/aa	eeAA/eeAa/eeaa

**Table 3:** Association of the KIT gene with white coat color traits.

Trait	Allele Symbol	Gene	Phenotype	References
Dominant White	W1–W35		White patterning or an entirely white coat with pink skin underneath	[36]
Tobiano	TO	KIT	Large markings on legs and small head markings	[37]
Sabino	SB1		A white spotting pattern with towering uneven stockings and a blaze on the face	[38]
Roan	RN		Dispersed white hair and dark points	[39]

**Table 4:** Mutant alleles in the *MITF* gene and their association with coat color phenotypes.

Gene	Allele	Mutation	Phenotype	Reference
MITF	SW1	10 bp insertion	Splashed white	[35]
MITF	SW3	Small deletion	Splashed white	[35]
MITF	SW5	63 kb deletion	White spotting, blue eyes	[94]
MITF	SW6	8.7 kb deletion	Splashed white, blue eyes	[95]
MITF	SW7	A novel three-base pair deletion	Splashed white	[96]
MITF	SW8	2.3 kb deletion	Splashed white, blue eyes	[97]
MITF	SW9	Missense mutation	Splashed white, blue eyes	[98]

## Data Availability

All the data are available in the manuscript.
